# Effect of Prenatal Glucocorticoid Exposure on Circadian Rhythm Gene Expression in the Brains of Adult Rat Offspring

**DOI:** 10.3390/cells11101613

**Published:** 2022-05-11

**Authors:** Alyssa Murray, Sujeenthar Tharmalingam, Sandhya Khurana, Christine Lalonde, Phong Nguyen, T. C. Tai

**Affiliations:** 1 Medical Sciences Division, NOSM University, 935 Ramsey Lake Rd., Sudbury, ON P3E 2C6, Canada; amurray@laurentian.ca (A.M.); sutharmalingam@nosm.ca (S.T.); skhurana@nosm.ca (S.K.); chrisnlalonde@gmail.com (C.L.); 2 Department of Biology, Laurentian University, Sudbury, ON P3E 2C6, Canada; shinmarizu@gmail.com; 3 Department of Chemistry and Biochemistry, Laurentian University, Sudbury, ON P3E 2C6, Canada; 4 Biomolecular Sciences Program, Laurentian University, Sudbury, ON P3E 2C6, Canada

**Keywords:** fetal programming, hypertension, spontaneously hypertensive rat (SHR), suprachiasmatic nucleus (SCN), amygdala, paraventricular nucleus

## Abstract

Circadian clocks control many vital aspects of physiology from the sleep-wake cycle to metabolism. The circadian clock operates through transcriptional-translational feedback loops. The normal circadian signaling relies on a ‘master clock’, located in the suprachiasmatic nucleus (SCN), which synchronizes peripheral oscillators. Glucocorticoid receptor (GR) signaling has the ability to reset the phase of peripheral clocks. It has been shown that maternal exposure to glucocorticoids (GCs) can lead to modification of hypothalamic-pituitary-adrenal (HPA) function, impact stress-related behaviors, and result in a hypertensive state via GR activation. We previously demonstrated altered circadian rhythm signaling in the adrenal glands of offspring exposed to the synthetic GC, dexamethasone (Dex). Results from the current study show that prenatal exposure to Dex affects circadian rhythm gene expression in a brain region-specific and a sex-specific manner within molecular oscillators of the amygdala, hippocampus, paraventricular nucleus, and prefrontal cortex, as well as the main oscillator in the SCN. Results also show that spontaneously hypertensive rats (SHR) exhibited dysregulated circadian rhythm gene expression in these same brain regions compared with normotensive Wistar-Kyoto rats (WKY), although the pattern of dysregulation was markedly different from that seen in adult offspring prenatally exposed to GCs.

## 1. Introduction

What is currently recognized as fetal programming was first postulated in the seminal works of Barker and Hales [1,2], who linked impaired gestational growth and development with an increased risk of several major diseases in later life [3,4,5]. The Barker hypothesis states that, under situations of maternal stress, many fetal organ systems and functions can undergo ‘programming’ *in utero*, which determines physiologic and metabolic responses in adult life [5,6]. There are many prenatal stressors that can induce negative programming and result in structural, physiologic, metabolic, and epigenetic changes in the offspring [7,8,9,10].

We have previously shown that the administration of synthetic glucocorticoids (GCs), such as dexamethasone (Dex) to Wistar-Kyoto (WKY) dams during the last trimester of pregnancy, results in offspring with increased blood pressure and elevated plasma epinephrine levels [7,8,11]. Subsequent whole-transcriptome analysis revealed that genes involved in circadian rhythm signaling are significantly dysregulated in the adrenal glands of adult animals exposed to Dex prenatally and that these animals display a circadian phase delay [12]. Interestingly, the Spontaneously Hypertensive Rat (SHR) (a genetic model of hypertension) also showed circadian disruption compared to their WKY counterparts; however, it was in the pattern of circadian phase advance [12].

Circadian rhythm clocks are regulators of the internal biological clock of the body. The body’s clock is trained to become activated by external stimuli, such as light [13], with nearly every cell in the body containing a molecular oscillator [14]. In mammals, the circadian system is organized into a master clock located in the suprachiasmatic nucleus (SCN) of the hypothalamus, which commands peripheral clocks located in almost every tissue of the body [15]. Light stimuli enter through the eyes and are registered by the cells of the SCN [13,16]. At the cellular level, transcription-translation feedback loops of the clock genes *Bmal1*, *Clock*, *Per1*, *Per2*, and *Per3*, as well as *Cry1* and *Cry2*, and their protein products result in the circadian oscillation of clock genes [13,14,16]. With diurnal stimuli, this negative feedback loop acts to maintain a rhythmic oscillation of gene expression that cycles approximately every 24 h and influences biological activities involved in the sleep/wake cycle, blood pressure, metabolism, and immune functioning [14,17]. Disruptions in circadian rhythms have been implicated in many diseases, such as breast cancer [18], obesity, type 2 diabetes [19,20], cardiometabolic disorders [21], and an overall higher risk of being immunocompromised [17]. Aside from damaging the circadian rhythm system through shifts in the diurnal sleep/wake cycle or an unbalanced stress hormone profile, circadian rhythms can also be disrupted through eating at unusual times [13], consuming alcohol, or taking antidepressants [22]. Misalignment of the circadian rhythm system can also have implications on behaviour, with mood disorders and other mental health problems likely impacted by the circadian cycle [23,24].

Under situations of stress, GCs are released from the adrenal gland through the activation of the hypothalamic-pituitary-adrenal (HPA) axis [16,25,26] and interact with the glucocorticoid receptor (GR), which is expressed in all tissues with notable exception of the SCN [27]. Once stimulated by GCs, the GR/GC complex can translocate to the nucleus and bind to glucocorticoid response elements (GREs) in the promoter regions of many genes, thereby influencing their expression [13]. The circadian rhythm genes *Per1* and *Per2* have GREs present in their promoter regions, allowing for transcriptional control of these rhythmic genes by the level of GCs in the body [27,28]. GCs are one of many hormones that are typically released based on circadian signaling and are under the control of the SCN [27]. The release of GC, mediated by stress, is capable of interacting with GR in cells other than the SCN, changing the expression levels of circadian genes, thereby causing shifts in the normal rhythms in the body [16,27]. Shifts in rhythms can occur through food restriction or the administration of exogenous GCs [29].

Limited research has been done surrounding the long-term effects of prenatal stress on the circadian rhythm profile of offspring and the potential implications that an adaptive programming of these crucial processes could have on overall health and disease. This investigation hypothesized that, due to the interplay of the circadian rhythm clocks of the body and GCs, prenatal GC exposure will result in dysregulated circadian rhythm gene expression in central and peripheral clocks of the offspring. To elucidate the mechanism behind circadian rhythm dysregulation in GC-exposed offspring, this study examined if GC exposure in utero can affect the expression of circadian rhythm genes in brain regions implicated in the regulation of the HPA axis at timepoint ZT4-ZT5.

This investigation also examined the mechanism of circadian rhythm dysregulation in brain regions implicated in HPA axis regulation for the WKY/SHR genetic model of hypertension through gene expression analysis. While it is known that circadian rhythm dysfunction poses an increased risk of hypertension, it is not known if the pathology of hypertension itself can lead to a dysregulated circadian rhythm. Blood pressure has its own rhythm in the body, and therefore, it was hypothesized that a disrupted blood pressure system would have an effect on circadian rhythm gene expression in these animals. Both the fetal programming and WKY/SHR models produce the same disease outcome of hypertension but have markedly different etiologies. Through investigating the expression of core clock genes in the brain of both hypertensive animal models, this study aims to identify the link between circadian rhythm gene dysregulation and the development of hypertension.

## 2. Experimental Procedure

### 2.1. Animal Care and Tissue Collection

WKY and SHR rats were purchased from Charles River Laboratory (Montreal, QC, Canada) and housed at Laurentian University’s animal care facility. All animal protocols were approved by the Laurentian University Animal Care Committee in accordance with guidelines from the Canadian Council on Animal Care, and this experiment was performed in alignment with the ARRIVE guidelines. Protocols regarding animal handling from delivery to the collection of tissues have been previously described [8,12]. Rats were exposed to a light-dark cycle of 12-h (light phase 6:00 a.m. to 6:00 p.m.) with food and water available ad libitum. The fetal programming of WKY rats with Dex was performed as previously reported [7,8]. Briefly, pregnant WKY females were administered subcutaneous injections of Dex (prepared in 0.9% NaCl with 4% ethanol) at 100 μg/kg/day or a saline solution control throughout the third trimester (days 15–21). The resulting offspring were weaned at 3 weeks and housed at 2–3 rats per cage according to sex. A separate cohort of 17-week-old male and female SHR and WKY rats were purchased and allowed to acclimate for 2 weeks. At 19 weeks of age, male and female WKY, SHR, and fetal programmed WKY rats were anesthetized through intraperitoneal injection of 75 mg ketamine and 5 mg xylazine per kg of body weight. Brains of each sex were collected from each cohort of animals, frozen on dry ice, and stored at −80 °C until further processing. Anesthetization and subsequent tissue collection occurred between 10 am and 11 am (Zeitgeber time, ZT4-ZT5).

### 2.2. Tissue Sectioning and Identification

All brain areas were dissected using sectioning or a micro-punch technique, as previously described [11]. Brains were mounted on a Cryostat blade (Leica Biosystems, Concord, ON, Canada) and thinly sliced. Each slice was then observed for neuroanatomical structures, and micro punctures were taken through the entirety of brain regions of interest [30]. Punctures from each brain area were combined in 2 mL microcentrifuge tubes and stored at −80 °C until time of extraction.

### 2.3. RNA Extraction from Brain Micro-punches

Total RNA was extracted from the SCN, paraventricular nucleus (PVN), amygdala, and hippocampus using the guanidine thiocyanate method [31], as previously described [11]. Briefly, 250 μL of an extraction solution (4 M guanidine thiocyanate, 25 mM sodium citrate, 0.5% sodium sarcosine, 0.1 M β-mercaptoethanol in distilled water), along with a stainless-steel bead was added to each tissue sample. Samples were homogenized using a TissueLyser (Qiagen, Montreal, QC, Canada) for 2 cycles of 1 min at 30 Hz. A total of 25 μL of 3 M sodium acetate (pH 4) was then added before an additional cycle of 1 min at 30 Hz on the TissueLyser. The supernatant was then mixed with 300 μL of a phenol/chloroform/isoamyl alcohol (25:24:1) solution before centrifuging at 12,000× *g* for 20 min. The aqueous phase was added to 1 mL of a 90% ethanol solution, and tubes were gently pulsed to mix and then placed at −80 °C for 4–5 days for RNA precipitation. Samples were then centrifuged at 12,000× *g* for 20 min at 4 °C before 250 μL of a 70% ethanol solution was added. An additional mixing and centrifugation step (12,000× *g* for 20 min at 4 °C) was undergone, and pellets were air-dried and dissolved in DEPC water before being placed on a Thermomixer (Eppendorf, Mississauga, ON, Canada) for 10 min at 37 °C.

### 2.4. RNA Extraction from Tissue Sections

Total RNA was extracted from the prefrontal cortex (PFC) using TRI reagent (Sigma-Aldrich, Oakville, ON, Canada), according to manufacturer’s instructions. Briefly, PFC brain regions were weighed and placed in a microcentrifuge tube with a stainless-steel bead and 1 mL TRI per 50 mg tissue. Tissue was homogenized using a TissueLyser for 2 cycles of 2 min at 30 Hz. The supernatant was mixed with chloroform (200 μL/1 mL TRI) and centrifuged at 12,000× *g* at 4 °C for 20 min. The aqueous layer containing RNA was mixed with isopropanol (250 μL/1 mL TRI) before centrifuging at 12,000× *g* for 8 min at 4 °C. The supernatant was discarded, and the remaining pellet was washed with a 70% ethanol solution (1 mL/1 mL TRI), before centrifuging at 7500× *g* for 5 min at 4 °C. Supernatant was removed, and the pellet was air-dried before being suspended in DEPC treated water and placed on a thermomixer for 10 min at 37 °C.

### 2.5. Spectrometry and cDNA Synthesis

RNA samples were analysed for concentration and relative purity with the NanoDrop One spectrophotometer (Thermo Fisher Scientific; Mississauga, ON, Canada). Potential genomic DNA contamination was removed from RNA samples through the use of the DNAseI kit (Sigma-Alrich, Oakville, ON, Canada), as per the manufacturer’s instructions. Following DNAse treatment, the RNA was reverse transcribed to cDNA using random primers, mixed dNTPs, and Mu-MLV RT (Promega, Madison, WI, USA), according to manufacturer’s instructions.

### 2.6. Reverse Transcribed Quantitative PCR (RT-qPCR)

All primers were selected, designed, and validated using the method previously described [12]. A complete list of validated primer sequences and corresponding information can be found in Supplementary Table S1. RT-qPCR analysis was performed using the QuantStudio 5 Real Time PCR machine (ThermoFisher Scientific, Mississauga, ON, Canada), with a total reaction volume of 15 μL. Each reaction contained 6 ng of the corresponding cDNA template, 600 nM of each forward and reverse primer pair, as well as the appropriate volume of 2X SYBR Green MasterMix (ThermoFisher Scientific, Mississauga, ON, Canada). Brain regions were run with mixed cycling parameters for 40–45 cycles. All samples were normalized to three brain-specific independent control housekeeping genes (*GAPDH*, *CycA*, and *Ywhaz*). Relative fold-change of the mRNA transcript level of each gene was calculated according to the ΔΔC_q_ method [32], with average ΔΔC_q_ and SEM calculated for each gene. If the C_q_ value was not detected prior to cycle number 40, this gene was considered not detected.

### 2.7. Statistics

Statistical analyses were performed using GraphPad Prism software (La Jolla, CA, USA). All data are presented as mean ± SEM (*n* = 8). Interquartile range (IQR) was used to determine outliers within groups. Statistical significance between two group (i.e., saline and Dex or WKY and SHR) was determined with an unpaired *t*-test. Values of *p* ≤ 0.05 were considered statistically significant.

## 3. Results

### 3.1. Mechanism of Circadian Rhythm Dysregulation in Offspring Prenatally Exposed to Glucocorticoids

Results from the RT-qPCR analysis of circadian rhythm genes involved in the core circadian loop across selected brain areas show dysregulated patterns of gene expression in offspring prenatally exposed to glucocorticoids compared to the saline controls, as summarized in Table 1. In the SCN, male offspring showed statistically significant differences in five of eight selected circadian genes: *Per1* (+3.5-fold), *Cry2* (+5.1-fold), *Per2* (−1.8-fold), *Per3* (−4.1-fold), and *Cry1* (−3.5-fold). In contrast, in the SCN of female offspring, *Clock* was the only gene affected, showing a decrease of 3.6-fold. In the hippocampus, males showed significant decrease in *Per1* (5.5-fold), as well as increase in *Per2* (1.9-fold) and *Cry2* (9.0-fold), while female offspring displayed statistically significant changes across three circadian rhythm genes: *Npas2* (−4.4-fold), *Clock* (+8.9-fold), and *Cry1* (+3.5-fold).

Results from the amygdala showed changes different from the dysregulation patterns seen in either the SCN or the hippocampus. Male offspring showed statistically significant changes in two circadian genes with decreases in *Per2* (8.3-fold) and *Cry2* (5.6-fold). Female offspring also displayed changes in two circadian genes, *Clock* (+5.3-fold) and *Per1* (+5.7-fold). It should also be noted that, while not all significant, females had the majority of genes showing an upregulation, while the males showed downregulation in all circadian genes analyzed. Within the PVN of GC-exposed offspring, males showed statistically significant increases in *Bmal1* (9.1-fold) and *Per2* (6.0-fold). Female offspring showed no statistically significant changes in the circadian genes analyzed. Lastly, analysis of circadian rhythm genes in the PFC revealed that expression levels in both male and female offspring were much more similar to their saline controls than any other brain area analyzed. Male offspring showed a statistically significant increase in *Per2* (1.3-fold); however, female offspring showed no statistically significant difference across the selected circadian genes.

### 3.2. Mechanism of Circadian Rhythm Dysregulation in the Spontaneously Hypertensive Rat

Gene expression analysis of circadian rhythm genes revealed dysregulated patterns across all brain areas analyzed in the SHR compared to the WKY controls, as summarized in Table 2. Within the SCN, female SHR showed a dysregulated pattern compared to their WKY controls, showing a decrease in *Bmal1* (7.4-fold) and an increase in *Per2* (3.1-fold). However, male offspring showed no statistically significant differences. In the hippocampus, females showed significant decrease in both *Per1* (7.4-fold) and *Cry2* (11.1-fold). Males showed a much greater amount of dysregulation in the hippocampus, with downregulations occurring in *Clock* (26.1-fold), *Per1* (126.5-fold), *Per2* (2.4-fold), *Cry1* (5.1-fold), and *Cry2* (7.1-fold). In the hippocampus, males and females had similar dysregulation patterns, with each gene showing a similar trend in fold change; however, males displayed a much greater change in expression levels.

Within the amygdala, females showed a great deal of dysregulation, with an overall trend of upregulation in the circadian panel and significance in *Bmal1* (26.1-fold), *Npas2* (21.2-fold), *Clock* (8.9-fold), *Per1* (9.9-fold), and *Per2* (11.1-fold). Males displayed a different pattern, with an increase in *Per1* (6.6-fold) and a decrease in *Cry2* (507-fold). Results from the PVN show significant change compared to their WKY controls. In females, significant increases were seen in *Bmal1* (4.8-fold), *Clock* (98.0-fold), *Per1* (7.1-fold), *Per2* (3.5-fold), and *Cry1* (5.5-fold). Males also showed widespread upregulation in the PVN region with significance in *Bmal1* (29.0-fold), *Npas2* (8.0-fold), *Clock* (11.7-fold), *Per2* (20.5-fold), *Cry1* (9.2-fold), and *Cry2* (15.2-fold). Finally, within the PFC, female offspring showed statistically significant increases in five genes: *Npas2* (3.3-fold), *Per1* (2.2-fold), *Per3* (2.4-fold), *Cry1* (1.5-fold), and *Cry2* (2.7-fold). Male offspring also showed significant changes with increased expression in *Npas2* (4.1-fold), *Per1* (2.3-fold), *Per3* (2.4-fold), and *Cry2* (2.3-fold) and a decrease in *Per1* (1.3-fold). Additionally, in this brain area, male and female dysregulation patterns were similar, with most genes displaying similar trends in fold change.

## 4. Discussion

This investigation examined the effect of prenatal exposure to the synthetic glucocorticoid, Dex, on central and peripheral circadian clocks in the brains of WKY rat offspring at ZT4-ZT5. It was determined that the core circadian genes demonstrated sex-specific expression and that dysregulation varied between brain areas. In the SCN, the core circadian oscillator, only *Clock* showed significant downregulation in females. Due to the integral role of Clock in circadian rhythm functioning and its close association with Bmal1, this downregulation was surprising. However, under Clock deficient conditions, the gene *Npas2* can be expressed to compensate and act as a substitute for Clock in the SCN [33]. Gene expression in the SCN of male animals showed greater dysregulation than the females, with five of eight genes dysregulated, indicating that males may exhibit a greater susceptibility to the effects of fetal programming than females. Coincidentally, the blood pressure phenotype of Dex-exposed male offspring had a more pronounced effect than females [7]. It has been shown that circulating estrogen has a protective role in maintaining daily circadian rhythms in females [34]. In this study, the male Dex-exposed animals exhibited a different and more dysregulated circadian pattern compared to females, and this may be due to the absence of the protective effects of estrogen in males. Unlike the females, males had normal expression levels in the positive limb of the oscillator; however, on the negative limb, significant dysregulation was seen. Erzberger and colleagues [35] hypothesized the redundancy of *Per* and *Cry* genes; therefore, gene expression increases in *Per1* (4.3-fold) and *Cry2* (5.1-fold) indicate that these genes drive the negative limb of the circadian oscillator, while decreases in *Per2* (1.8-fold), *Per3* (4.1-fold), and *Cry1* (3.5-fold) indicate these genes play a lesser role.

In the hippocampus, females displayed the greatest dysregulation at the positive limb of the oscillator. While it was previously thought that Npas2 had a SCN-specific role, it has been shown that peripheral circadian clocks are fundamentally similar and therefore share this Npas2/Clock compensation mechanism [33]. Since *Clock* is significantly upregulated, a downregulation in *Npas2*, as shown in this study, would be expected. In males, dysregulation in the negative limb was seen. The hippocampal region of the brain is extremely dense with GRs [36]. Both Cry1 and Cry2 are capable of interacting with GR and altering the transcriptional response to GCs, which can result in high levels of circulating GCs and suppression of the HPA axis [37] through nerve cell damage and compromised hippocampal function [38]. In the GC-exposed males, this large increase in *Cry2* expression may play a protective role by inhibiting the activation of GR. Consequently, the large decrease in *Per1* may follow from the effect of *Cry2*, as GR will no longer be activated and thereby will be incapable of translocating to the nucleus to interact with GREs, resulting in reduced *Per1* transcription [28].

In the amygdala, females displayed dysregulation of *Clock* while *Bmal1* was not affected. This result was surprising given that the positive limb of the oscillator has evolved to activate simultaneously with Clock and Bmal1, forming a heterodimer to activate circadian driven genes [39]. Males, on the other hand, showed downregulation of all circadian genes, particularly *Per2* (8.3-fold) and *Cry2* (5.6-fold). This significantly dysregulated system, with all genes being downregulated, indicates that the normal cycling pattern of the circadian system is not being conserved in males compared to females [34]. Redundancy in the *Per* and *Cry* genes ensure a narrow range of entrainment of the circadian clocks, with *Per2/Cry2* mutants shown to be less sensitive to rhythmicity and exhibiting a wider range of entrainment [35]. Thus, the lowered expression of *Per2* and *Cry2* may be acting to compensate for the dysregulation seen in the amygdala of these animals.

In the paraventricular nucleus, an important region in the hypothalamus for autonomic control [40], a sex-specific pattern of dysregulation could be seen with no changes in females, while males showed widespread upregulation in both limbs of the circadian oscillator. It has been reported that circadian rhythm expression profiles of various clock genes are similar between the SCN and PVN in male Wistar rats [41]. This is anticipated as the PVN acts as a relay between the SCN and the HPA axis. However, in this system, the pattern of circadian gene expression is extremely different in the PVN compared to the SCN. This points to either a delay in rhythm between the SCN and PVN or a neural miscommunication between these closely connected brain areas. Finally, the prefrontal cortex, which regulates thoughts, actions, emotions, and other higher order cognition [42], showed the least amount of change compared to saline controls in both sexes.

Examining the five brain areas analyzed, as well as the adrenal glands of male offspring [12], a few trends can be seen. Overall, trends show that female offspring are more protected from circadian rhythm dysregulation. When dysregulation did occur, the female animals tend to display more dysregulation in the positive limb of the oscillator, while in the males, there is a disruption of the negative limb. Interestingly, while females exhibited the most dysregulation in the positive limb, *Bmal1* was never affected. In contrast, in the males, *Bmal1* was the only gene in the positive limb to be dysregulated. Based on clinical SNP data on *Bmal1*, strong associations have been found between *Bmal1* SNPs that confer reduced transcriptional efficiency and increased susceptibility to type 2 diabetes and hypertension [43]. The dysfunctional *Bmal1* expression in males could cause downstream effects on the entire circadian loop, which may contribute to the hypertensive phenotype that is seen in both this model, as well as in the *Bmal1* knockout model [12,44].

Additionally, when comparing the male brain areas to the adrenal glands, it is clear that a more significant dysregulation occurred in the adrenal glands. While the male adrenal glands show a clear pattern of dysregulation that implies a shift in phase from the saline controls [12], the patterns in the brain tend to be much more erratic, with no clear dysregulation mechanism. This also holds true within the different brain areas examined. In addition, sex-specific effects were seen on the positive and negative limbs of the oscillator; however, these effects were not consistent. Overall, these findings suggest that Dex-exposed animals have an uncoupling of the circadian rhythm in the peripheral clocks, such as the adrenal gland and other brain structures from the central clock in the SCN. Since the offspring were exposed to high levels of GCs *in utero*, it is possible that mistimed GC administration permanently uncoupled the peripheral clocks from the central clock through GR activation and subsequent signaling [27].

The brain regions all show a different pattern of dysregulation, and the expected reciprocal expression of genes in the positive and negative limbs of the oscillator are not seen. This suggests these brain regions do not have a properly cycling circadian system. While we and others have shown that peripheral clocks can be uncoupled from the SCN and still maintain an independent circadian rhythm [12,45], the expression seen in the brain regions suggest that they are not maintaining a cycling circadian rhythm in general. At this point, it is unclear which clock system (central or peripheral) was the initiator of this widespread dysregulation or whether this programmed dysregulation occurred simultaneously across all clocks.

Circadian rhythm function in central and peripheral clocks was also investigated at ZT4-ZT5 in the SHR genetic model of hypertension. These results also showed that circadian genes demonstrated sex-specific expression, varied between brain areas, and had dysregulation patterns that were markedly different than those seen in the GC-exposed animals. In the SCN, SHR males showed no dysregulation, which is interesting given what was seen in the Dex model. It appears that the central clock in male SHR animals is fully functional. SHR females, on the other hand, showed changes in *Bmal1* (−7.4-fold) and *Per2* (+3.1-fold). It is known that there is a SNP in the *Bmal1* gene of SHR animals [43], which could account for the decrease seen in *Bmal1* expression.

In the hippocampus, SHR males showed significant dysregulations in both the positive and negative limbs. Here, *Npas2* and *Bmal1* are likely driving the positive limb of the circadian system, since *Clock* expression was downregulated (26.1-fold). Similarly, several core circadian genes are downregulated in the negative limb of the oscillator. While Npas2 and Clock have been found to have similar circadian roles [33], it is unclear whether the Clock/Bmal1 complex can activate components of the negative limb that the Npas2/Bmal1 complex cannot, and vice versa. For example, the transcription factor DBP, which is regulated by Clock/Bmal1, can cooperatively activate *Per1* with the Clock/Bmal1 complex by directly binding to its promoter region [46]. Perhaps DBP was not activated in the system due to the downregulation of *Clock*, which in turn resulted in the downregulation of *Per1*. In the hippocampus of the female SHR, there was downregulation of nearly all circadian genes with statistical significance in *Per1* (7.4-fold) and *Cry2* (11.1-fold). The visual pattern of dysregulation in male and female hippocampi is very similar, though the fold changes are larger in males. It seems that the circadian machinery in the hippocampus of SHR are repressed compared to their WKY counterparts in both sexes. A study investigating the effects of circadian rhythm disorder (CRD) on the hippocampus of SHR and WKY rats found that CRD and hypertension reduced memory performance and caused changes in hippocampal plasticity by decreasing the number of neurons and astrocytes and reduced blood flow in the brain [47]. It appears that the hippocampus is particularly vulnerable to the effects of CRD and hypertension, which could explain the high level of circadian dysregulation in the brain structure, as observed in this study.

In the amygdala of female SHR, nearly all circadian genes showed an upregulation, which is the opposite of what was seen in the hippocampus. It seems, in the amygdala, the circadian clock is activated in SHRs compared to female WKY. Given the role of the amygdala in fear and the fight or flight response, it is possible that an overactive amygdala is contributing to the hypertensive phenotype of female SHR. The amygdala normally reinforces emotionally-induced defensive reactions, and when the amygdala of SHRs is lesioned, blood pressure is significantly attenuated [48]. It is thought that the amygdala may play an important role in aggravating hypertension in SHR animals by reinforcing maladaptive reactions to stressful situations [48]. In the amygdala of male SHRs, there was a significant upregulation of *Per1* (6.6-fold) and downregulation of *Cry2* (507-fold). Due to the redundant nature of the *Per* and *Cry* genes [35], it is possible that the dysregulation seen in this brain region is the result of Per1 and Cry2 not being used in the circadian loop compared to the other Pers and Cry1.

In the PVN of both male and female SHR, all circadian genes were upregulated to some extent. This is the opposite of what was seen in the hippocampus of SHRs and presents another brain region, where male and female data are paralleled. Like in the female amygdala, it appears the circadian clock machinery is activated compared to WKYs. It has been suggested that the PVN participates in blood pressure regulation, and through lesioning of the PVN in SHR and WKY rats, it has been shown that the PVN contributes to the development of spontaneous hypertension [49]. The activation of the core circadian rhythm genes in the PVN may be contributing to the blood pressure phenotype seen in SHR animals. Finally, a similar pattern of dysregulation was seen in the PFC of the male and female SHR. Both males and females displayed increased *Npas2* with 4.1-fold in males and 3.3-fold in females. SNP variations in *Npas2* have been shown to be linked to hypertension in humans [50]. Variations in the *Per*s and *Cry*s could be related to an altered timing compared to the WKYs. As discussed in our previous work, SHR animals have a phase advance with regard to circadian rhythms [12]. Perhaps the upregulation of the negative limb and downward trend in the positive limb are accounting for this alteration in circadian signaling.

Examining all five brain areas, in addition to the adrenal glands of the male offspring [12], we can see that male and female SHR have dysregulation occurring in all brain areas and the patterns of dysregulation in the hippocampus, PVN, and PFC were very similar in both sexes. The dysregulation was widespread across the circadian oscillator, and no clear mechanism could link the brain areas together. Over-activation of the circadian system in the PVN of these animals may have a link to the hypertensive phenotype; however, further investigation is required. When comparing the male brain areas to the adrenal gland data [12], while the SHR adrenals show a clear shift from their WKY counterparts, the brain areas appear to be more dysregulated overall. Given that the adrenal glands are indicative of a phase shift from the WKY controls, perhaps the brain areas are also part of a shift in rhythm but different from the adrenal gland. In conclusion, the current study shows a notable tissue-specific circadian dysregulations across the five brain regions and a sex-specific effect in only two brain regions.

Differences in circadian rhythms between males and females have been noted in the literature. As previously mentioned in the discussion of the differences between males and females in the SCN, estrogen has been shown to have a protective role in maintaining the circadian rhythms of females [34]. This protective effect may be more necessary in maintaining the normal circadian rhythm functioning in females, as circadian rhythm disruption leads to more detrimental outcomes in females compared to males [51]. Female circadian rhythms are less likely to be shifted from normal cycling patterns; however, when they are shifted, they produce a much greater effect and can lead to significant mental health disorders [51]. In a study involving the human cerebral cortex, it was reported that the circadian cycles in men and women run differently, with rhythms in men showing a slight delay in comparison to females [52]. The absence of the *Per1* gene elicits a different response between the sexes: in male mice, a lack of *Per1* disrupted circadian blood pressure rhythms; however, they were maintained in female mice [53]. Finally, women tend to have less circadian disruption overall [34], and this is in accordance with what was found in this investigation, particularly in the Dex model.

It should be noted that circadian rhythms function over a 24-h period and that the expression of circadian genes rise and fall throughout this cycle. While this study only looked at one point in time (ZT4-ZT5), this does not negate the significant changes in circadian gene expression that were identified in this study. We have previously discovered similar alterations in circadian genes in the adrenal glands of Dex model offspring at the same single time point (ZT4-ZT5) [12]. However, it would be interesting to profile changes in circadian rhythm gene expression within various brain regions over a 24-h period to further corroborate the findings from this study.

In conclusion, these results show the impact of sex as a biological variable that plays a central role in physiological adaptation and the outcomes that arise from exposure to prenatal stress. While both male and female offspring were subjected to the same conditions *in utero*, the subsequent effects seen were extremely divergent. This study was the first to examine the molecular mechanism of circadian rhythm gene dysregulation in the brains of fetal programmed offspring where this dysregulation was found to be both sex- and tissue-specific. This investigation also investigated SHR animals, where dysregulation was found to be tissue-specific but not sex-specific with the hippocampus, PVN, and PFC, showing similarities between sexes. This was the first study to examine all core circadian clock genes across these brain regions in SHRs. These findings provide a deeper understanding of the connection between fetal programming, circadian rhythms, and blood pressure and also highlights the importance of studying sex-specific differences within animal models.

## Figures and Tables

**Table 1:** Summary of circadian rhythm gene expression changes in Dex exposed offspring. Offspring exposed to Dex *in utero* were sacrificed at 19 weeks of age between 10 a.m. and 11 a.m. (ZT4-ZT5). RNA was isolated from brain regions following sectioning or micro-punching. Gene expression was detected through RT-qPCR and analyzed according to the ΔΔC_q_ method. Expression is represented as mean fold change (± SEM) of the Dex animals relative to saline control. Statistically significant (*p* < 0.05) changes compared to saline are represented as bolded red text (upregulation) and blue text (downregulation). If the C_q_ value was detected past cycle number 40, these genes were considered not detected.

Sex	Gene	SCN	Hippocampus	Amygdala	PVN	PFC
Male	*Bmal1*	−1.5 ± 1.4	+3.2 ± 2.3	−1.5 ± 1.9	**+9.1 ± 8.2**	+1.3 ± 0.5
	*Npas2*	Not detected	+1.8 ± 2.3	−3.8 ± 3.5	+2.0 ± 3.4	−1.2 ± 0.1
	*Clock*	−2.6 ± 1.3	−1.3 ± 0.9	−2.4 ± 1.4	+1.6 ± 1.8	+1.3 ± 0.4
	*Per1*	**+4.3 ± 1.8**	**−5.5 ± 3.0**	−1.9 ± 0.8	+1.7 ± 1.7	+1.1 ± 0.1
	*Per2*	**−1.8 ± 0.3**	**+1.9 ± 0.5**	**−8.3 ± 6.4**	**+6.0 ± 4.9**	**+1.3 ± 0.1**
	*Per3*	**−4.1 ± 2.3**	Not detected	−2.2 ± 1.8	−5.6 ± 9.2	+1.2 ± 0.2
	*Cry1*	**−3.5 ± 1.1**	−1.3 ± 0.5	−1.6 ± 1.1	+3.5 ± 2.4	−1.2 ± 0.3
Female	*Bmal1*	−1.2 ± 1.4	+2.5 ± 2.2	−1.3 ± 1.0	−1.4 ± 0.7	+1.3 ± 0.2
	*Npas2*	+1.9 ± 3.2	**−4.4 ± 0.6**	+1.9 ± 1.9	−1.0 ± 0.9	−1.1 ± 0.1
	*Clock*	**−3.6 ± 1.9**	**+8.9 ± 5.2**	**+5.3 ± 3.8**	+2.7 ± 1.5	+1.2 ± 0.2
	*Per1*	−1.6 ± 0.8	+2.0 ± 1.7	**+5.7 ± 3.3**	+1.1 ± 1.2	−1.3 ± 0.2
	*Per2*	+1.0 ± 0.2	+1.4 ± 0.5	1.0 ± 0.6	−1.2 ± 0.8	+2.1 ± 0.8
	*Per3*	−1.5 ± 1.1	+1.2 ± 1.9	−1.3 ± 0.8	Not detected	+1.3 ± 0.2
	*Cry1*	+1.0 ± 0.6	**+3.5 ± 1.5**	+1.3 ± 1.1	−2.2 ± 3.0	+1.1 ± 0.2
	*Cry2*	−1.5 ± 0.7	+1.2 ± 1.1	+2.0 ± 2.1	−1.0 ± 0.5	−1.0 ± 0.1

**Table 2:** Summary of circadian rhythm gene expression changes in the spontaneously hypertensive rat. Offspring exposed to Dex *in utero* were sacrificed at 19 weeks of age between 10 a.m. and 11 a.m. (ZT4-ZT5). RNA was isolated from brain regions following sectioning or micro-punching. Gene expression was detected through RT-qPCR and analyzed according to the ΔΔC_q_ method. Expression is represented as the mean fold change (± SEM) of SHR animals relative to the WKY control. Statistically significant (*p* < 0.05) changes compared to WKY are represented as bolded red text (upregulation) and blue text (downregulation). If the C_q_ value was detected past cycle number 40, these genes were considered not detected.

Sex	Gene	SCN	Hippocampus	Amygdala	PVN	PFC
Male	*Bmal1*	+1.0 ± 0.9	−4.2 ± 3.1	−3.5 ± 4.5	**+29.0 ± 20.5**	−1.2 ± 0.2
	*Npas2*	Not detected	−1.4 ± 1.3	+7.5 ± 7.8	**+8.0 ± 7.2**	**+4.1 ± 0.5**
	*Clock*	−4.6 ± 3.2	**−26.1 ± 11.9**	+1.6 ± 1.5	**+11.7 ± 11.1**	−1.0 ± 0.1
	*Per1*	+3.6 ± 3.2	**−126.5 ± 99.0**	**+6.6 ± 3.7**	+1.6 ± 1.2	**+2.3 ± 0.1**
	*Per2*	1.1 ± 0.4	**−2.4 ± 0.9**	−1.1 ± 0.9	**+20.5 ± 6.8**	**−1.3 ± 0.1**
	*Per3*	Not detected	−5.1 ± 5.9	Not detected	+7.6 ± 14.6	**+2.4 ± 0.3**
	*Cry1*	−1.0 ± 1.3	**−5.1 ± 3.7**	−1.4 ± 1.4	**+9.2 ± 12.9**	−1.2 ± 0.1
	*Cry2*	−1.6 ± 0.9	**−7.1 ± 4.5**	**−507 ± 448**	**+15.2 ± 13.8**	**+2.3 ± 0.1**
Female	*Bmal1*	**−7.4 ± 7.1**	−2.2 ± 1.9	**+26.1 ± 21.4**	**+4.8 ± 2.5**	−1.2 ± 0.1
	*Npas2*	Not detected	−1.6 ± 1.3	**+21.2 ± 13.4**	+4.4 ± 6.0	**+3.3 ± 0.2**
	*Clock*	+1.4 ± 1.0	−6.9 ± 9.6	**+8.9 ± 5.6**	**+98.0 ± 81.8**	−1.1 ± 0.1
	*Per1*	−2.1 ± 1.1	**−7.4 ± 6.8**	**+9.9 ± 7.6**	**+7.1 ± 4.6**	**+2.2 ± 0.3**
	*Per2*	**+3.1 ± 1.5**	−1.6 ± 1.1	**+11.1 ± 5.7**	**+3.5 ± 1.7**	−1.1 ± 0.1
	*Per3*	−1.0 ± 1.9	+1.9 ± 3.3	+2.8 ± 4.2	Not detected	**+2.4 ± 0.4**
	*Cry1*	−1.2 ± 0.9	−3.5 ± 4.3	**+13.1 ± 12.9**	**+5.5 ± 3.4**	**+1.5 ± 0.2**
	*Cry2*	+1.1 ± 1.0	**−11.1 ± 10.5**	−1.7 ± 0.6	+1.5 ± 0.8	**+2.7 ± 0.1**

## Data Availability

All relevant data are within the manuscript.

## Supplementary Materials

The following are available online at www.mdpi.com/article/10.3390/cells11101613/s1, Table S1: Primer sequences used for RT-qPCR with amplicon size and annealing temperatures.

## References

[1] Barker D.J.P. (1990). The Fetal and Infant Origins of Adult Disease: The Womb May Be More Important than the Home. BMJ.

[2] Hales C.N., Barker D.J.P., Clark P.M.S., Cox J., Fall C., Oxmond C., Winter P.D. (1991). Fetal and Infant Growth and Impaired Glucose Tolerance at Age 64. BMJ.

[3] Barker D.J.P. (1995). Fetal Origins of Coronary Heart Disease. BMJ.

[4] Godfrey K.M., Barker D.J.P. (2001). Fetal Programming and Adult Health. Public Health Nutr..

[5] Godfrey K.M., Barker D.J.P. (2000). Fetal Nutrition and Adult Disease. Am. J. Clin. Nutr..

[6] Barker D.J.P. (1995). The Fetal and Infant Origins of Disease. Eur. J. Clin. Investig..

[7] Khurana S., Grandbois J., Tharmalingam S., Murray A., Graff K., Nguyen P., Tai T.C. (2019). Fetal Programming of Adrenal PNMT and Hypertension by Glucocorticoids in WKY Rats Is Dose and Sex-Dependent. PLoS ONE.

[8] Nguyen P., Khurana S., Peltsch H., Grandbois J., Eibl J., Crispo J., Ansell D., Tai T.C. (2015). Prenatal Glucocorticoid Exposure Programs Adrenal PNMT Expression and Adult Hypertension. J. Endocrinol..

[9] Lamothe J., Khurana S., Tharmalingam S., Williamson C., Byrne C.J., Khaper N., Mercier S., Tai T.C., Moorthy B. (2020). The Role of DNMT and HDACs in the Fetal Programming of Hypertension by Glucocorticoids. Oxidative Med. Cell. Longev..

[10] Lau C., Rogers J.M., Desai M., Ross M.G. (2011). Fetal Programming of Adult Disease: Implications for Prenatal Care. Obstet. Gynecol..

[11] Grandbois J., Khurana S., Graff K., Nguyen P., Meltz L., Tai T.C. (2016). Phenylethanolamine N-Methyltransferase Gene Expression in Adrenergic Neurons of Spontaneously Hypertensive Rats. Neurosci. Lett..

[12] Tharmalingam S., Khurana S., Murray A., Lamothe J., Tai T.C. (2020). Whole Transcriptome Analysis of Adrenal Glands from Prenatal Glucocorticoid Programmed Hypertensive Rodents. Sci. Rep..

[13] Koch C.E., Leinweber B., Drengberg B.C., Blaum C., Oster H. (2017). Interaction between Circadian Rhythms and Stress. Neurobiol. Stress.

[14] Zelinski E.L., Deibel S.H., McDonald R.J. (2014). The Trouble with Circadian Clock Dysfunction: Multiple Deleterious Effects on the Brain and Body. Neurosci. Biobehav. Rev..

[15] Nakao A., Nakamura Y., Shibata S. (2015). The Circadian Clock Functions as a Potent Regulator of Allergic Reaction. Allergy Eur. J. Allergy Clin. Immunol..

[16] Nader N., Chrousos G.P., Kino T. (2010). Interactions of the Circadian CLOCK System and the HPA Axis. Trends Endocrinol. Metab..

[17] Lange T., Dimitrov S., Born J. (2010). Effects of Sleep and Circadian Rhythm on the Human Immune System. Ann. N. Y. Acad. Sci..

[18] Stevens R.G. (2005). Circadian Disruption and Breast Cancer: From Melatonin to Clock Genes. Epidemiology.

[19] Shi S., Ansari T.S., Mcguinness O.P., Wasserman D.H., Johnson C.H. (2013). Circadian Disruption Leads to Insulin Resistance and Obesity. Curr. Biol..

[20] Huang W., Marcheva B., Bass J., Huang W., Ramsey K.M., Marcheva B., Bass J. (2011). Circadian Rhythms, Sleep, and Metabolism. J. Clin. Investig..

[21] McGinnis G.R., Young M.E. (2016). Circadian Regulation of Metabolic Homeostasis: Causes and Consequences. Nat. Sci. Sleep.

[22] Rosenwasser A.M. (2001). Alcohol, Antidepressants, and Circadian Rhythms: Human and Animal Models. Alcohol Res. Health.

[23] Salvatore P., Indic P., Murray G., Baldessarini R.J. (2012). Biological Rhythms and Mood Disorders. Dialogues Clin. Neurosci..

[24] McEwen B.S., Bowles N.P., Gray J.D., Hill M.N., Hunter R.G., Karatsoreos I.N., Nasca C. (2016). Mechanism of Stress in the Brain. Nat. Neurosci..

[25] Drake A.J., Tang J.I., Nyirenda M.J. (2007). Mechanisms Underlying the Role of Glucocorticoids in the Early Life Programming of Adult Disease. Clin. Sci..

[26] Nicolaides N.C., Charmandari E., Chrousos G.P., Kino T. (2014). Circadian Endocrine Rhythms: The Hypothalamic-Pituary-Adrenal Axis and Its Actions. Ann. N. Y. Acad. Sci..

[27] Dickmeis T. (2009). Glucocorticoids and the Circadian Clock. J. Endocrinol..

[28] Yan J., Wang H., Liu Y., Shao C. (2008). Analysis of Gene Regulatory Networks in the Mammalian Circadian Rhythm. PLoS Comput. Biol..

[29] Balsalobre A., Brown S.A., Marcacci L., Tronche F., Kellendonk C., Reichardt H.M., Schutz G., Schibler U. (2000). Resetting of Circadian Time in Peripheral Tissues by Glucocorticoid Signaling. Science.

[30] Paxinos G., Watson C. (2006). The Rat Brain in Stereotaxic Coordinates.

[31] Chomczynski P., Sacchi N. (1987). Single-Step Method of RNA Isolation by Acid Guanidinium Thiocyanate-Phenol-Chloroform Extraction. Anal. Biochem..

[32] Livak K.J., Schmittgen T.D. (2001). Analysis of Relative Gene Expression Data Using Real-Time Quantitative PCR and the 2^−ΔΔCT^ Method. Methods.

[33] Landgraf D., Wang L.L., Diemer T., Welsh D.K. (2016). NPAS2 Compensates for Loss of CLOCK in Peripheral Circadian Oscillators. PLoS Genet..

[34] Palmisano B.T., Stafford J.M., Pendergast J.S. (2017). High-Fat Feeding Does Not Disrupt Daily Rhythms in Female Mice Because of Protection by Ovarian Hormones. Front. Endocrinol..

[35] Erzberger A., Hampp G., Granada A.E., Albrecht U., Herzel H. (2013). Genetic Redundancy Strengthens the Circadian Clock Leading to a Narrow Entrainment Range. J. R. Soc. Interface.

[36] Jeanneteau F., Chao M.V. (2013). Are BDNF and Glucocorticoid Activities Calibrated?. Neuroscience.

[37] Lamia K.A., Papp S.J., Yu R.T., Barish G.D., Uhlenhaut N.H., Jonker J.W., Downes M., Evans R.M. (2011). Cryptochromes Mediate Rhythmic Repression of the Glucocorticoid Receptor. Nature.

[38] Sapolsky R.M., Meaney M.J. (1986). Maturation of the Adrenocortical Stress Response: Neuroendocrine Control Mechanisms and the Stress Hyporesponsive Period. Brain Res. Rev..

[39] Lee Y., Lee J., Kwon I., Nakajima Y., Ohmiya Y., Son G.H., Lee K.H., Kim K. (2010). Coactivation of the CLOCK-BMAL1 Complex by CBP Mediates Resetting of the Circadian Clock. J. Cell Sci..

[40] Ferguson A.V., Latchford K.J., Sanon W.K. (2008). The Paraventricular Nucleus of the Hypothalamus—A Potential Target for Integrative Treatment of Autonomic Dysfunction. Expert Opin. Ther. Targets.

[41] Asai M., Yoshinobu Y., Kaneko S., Mori A., Nikaido T., Moriya T., Akiyama M., Shibata S. (2001). Circadian Profile of per Gene MRNA Expression in the Suprachiasmatic Nucleus, Paraventricular Nucleus, and Pineal Body of Aged Rats. J. Neurosci. Res..

[42] Arnsten A.F.T. (2009). Stress Signalling Pathways That Impair Prefrontal Cortex Structure and Function. Nat. Rev. Neurosci..

[43] Woon P.Y., Kaisaki P.J., Bragança J., Bihoreau M.T., Levy J.C., Farrall M., Gauguier D. (2007). Aryl Hydrocarbon Receptor Nuclear Translocator-like (BMAL1) Is Associated with Susceptibility to Hypertension and Type 2 Diabetes. Proc. Natl. Acad. Sci. USA.

[44] Marcheva B., Ramsey K.M., Peek C.B., Affinati A., Maury E., Bass J. (2013). Circadian Clocks and Metabolism. Handb. Exp. Pharmacol..

[45] Yoo S.-H., Yamazaki S., Lowrey P.L., Shimomura K., Ko C.H., Buhr E.D., Siepka S.M., Hong H.-K., Oh W.J., Yoo O.J. (2004). PERIOD2: LUCIFERASE Real-Time Reporting of Circadian Dynamics Reveals Persistent Circadian Oscillations in Mouse Peripheral Tissues. Proc. Natl. Acad. Sci. USA.

[46] Yamaguchi S., Mitsui S., Yan L., Yagita K., Miyake S., Okamura H. (2000). Role of DBP in the Circadian Oscillatory Mechanism. Mol. Cell. Biol..

[47] Wang Y.L., Zhang Y.G., Wang W.Z., Liu X., Chi Y.F., Lei J.F., Zhang B.G., Zhang T. (2020). Effects of Circadian Rhythm Disorder on the Hippocampus of SHR and WKY Rats. Neurobiol. Learn. Mem..

[48] Folkow B., Hallback-Norlander M., Martner J., Nordborg C. (1982). Influence of Amygdala Lesions on Cardiovascular Responses to Alerting Stimuli, on Behaviour and on Blood Pressure Development in Spontaneously Hypertensive Rats. Acta Physiol. Scand..

[49] Takeda K., Nakata T., Takesako T., Itoh H., Hirata M., Kawasaki S., Hayashi J., Oguro M., Sasaki S., Nakagawa M. (1991). Sympathetic Inhibition and Attenuation of Spontaneous Hypertension by PVN Lesions in Rats. Brain Res..

[50] Englund A., Kovanen L., Saarikoski S.T., Haukka J., Reunanen A., Aromaa A., Lönnqvist J., Partonen T. (2009). NPAS2 and PER2 Are Linked to Risk Factors of the Metabolic Syndrome. J. Circadian Rhythm..

[51] Chun L.E., Woodruff E.R., Morton S., Hinds L.R., Spencer R.L. (2015). Variations in Phase and Amplitude of Rhythmic Clock Gene Expression across Prefrontal Cortex, Hippocampus, Amygdala, and Hypothalamic Paraventricular and Suprachiasmatic Nuclei of Male and Female Rats. J. Biol. Rhythm..

[52] Lim A.S.P., Myers A.J., Yu L., Buchman A.S., Duffy J.F., De Jager P.L., Bennett D.A. (2013). Sex Difference in Daily Rhythms of Clock Gene Expression in the Aged Human Cerebral Cortex. J. Biol. Rhythm..

[53] Douma L.G., Solocinski K., Holzworth M.R., Crislip G.R., Masten S.H., Miller A.H., Cheng K.-Y., Lynch I.J., Cain B.D., Wingo C.S. (2018). Female C57BL/6J Mice Lacking the Circadian Clock Protein PER1 Are Protected from Nondipping Hypertension. Am. J. Physiol. Regul. Integr. Comp. Physiol..

[54] Murray A. (2021). Effects of Prenatal Stress on Circadian Rhythms and Metabolism in Adult Rat Offspring. Master’s Thesis.

