# Supplementary material for: Effect of Prenatal Glucocorticoid Exposure on Circadian Rhythm Gene Expression in the Brains of Adult Rat Offspring
Source: Cells. 2022 May 11;11(10):1613. doi: 10.3390/cells11101613 (PMC9139626; doi:10.3390/cells11101613)
Supplement: Supplementary file 1 [file cells-11-01613-s001.zip › cells-1678765-supplementary.pdf]

## Supplementary Information

**Supplementary Table S1.** Primer sequences used for RT-qPCR with amplicon size and annealing temperatures

| Gene/ Accession ID | Sequence (5'-3') | Amplicon Size (bp) | Annealing Temp (°C) |
|---|---|---|---|
| *Bmal1* | TGCCACTGACTACCAAGAAAGT | 138 | 60 |
| NM_024362.2 | ATTTTGTCCCGACGCCTCTT | | |
| *Npas2* | TCTTCTGAGAGGCAGCTTGAA | 85 | 60 |
| NM_001108214.2 | CAGGAGGGGCTAGGCACATT | | |
| *Clock* | AAGATGACACAGCGGAGGTC | 127 | 60 |
| NM_021856.2 | ACTGTGACATGCCTTGTGGG | | |
| *Per1* | CTCTCCGCAACCAGGATACC | 139 | 60 |
| NM_001034125.1 | GCTAGGAGCTCTGAGAAGCG | | |
| *Per2* | AAGTGACGGGTCGAGCAAAG | 71 | 60 |
| NM_031678.1 | CATGTCGGGCTCTGGAATGA | | |
| *Per3* | CCACCCTCTCCAGGTCATGT | 125 | 60 |
| NM_023978.2 | CGCCACTGAAACCAAAACCAA | | |
| *Cry1* | CCCACTAAAGCAAGGAAGAAGC | 134 | 60 |
| NM_198750.2 | CCCGCATGCTTTCGTATCAGTT | | |
| *Cry2* | GGACTACATCCGGCGATACC | 112 | 60 |
| NM_133405.2 | GCCAATGATGCACTTAGCGG | | |
